# Empirical dynamic modelling and enhanced causal analysis of short-length *Culex* abundance timeseries with vector correlation metrics

**DOI:** 10.1038/s41598-024-54054-4

**Published:** 2024-02-13

**Authors:** Nikos Kollas, Sandra Gewehr, Ioannis Kioutsioukis

**Affiliations:** 1 Department of Physics, University of Patras, 26504 Patras, Greece; 2 Ecodevelopment S.A., 57010 Filyro, Greece

**Keywords:** Environmental impact, Population dynamics, Mathematics and computing

## Abstract

Employing Empirical Dynamic Modelling we investigate whether model free methods could be applied in the study of *Culex* mosquitoes in Northern Greece. Applying Simplex Projection and S-Map algorithms on yearly timeseries of maximum abundances from 2011 to 2020 we successfully predict the decreasing trend in the maximum number of mosquitoes which was observed in the rural area of Thessaloniki during 2021. Leveraging the use of vector correlation metrics we were able to deduce the main environmental factors driving mosquito abundance such as temperature, rain and wind during 2012 and study the causal interaction between neighbouring populations in the industrial area of Thessaloniki between 2019 and 2020. In all three cases a chaotic and non-linear behaviour of the underlying system was observed. Given the health risk associated with the presence of mosquitoes as vectors of viral diseases these results hint to the usefulness of EDM methods in entomological studies as guides for the construction of more accurate and realistic mechanistic models which are indispensable to public health authorities for the design of targeted control strategies and health prevention measures.

## Introduction

One of the many negative side effects of climate change, among others, is the increase in the number of zoonotic viral diseases transmitted through animal hosts^1^. A notable example of this is the common house mosquito (*Culex* spp.). Several species of the genus *Culex* are known to be principal vectors that can transmit diseases such as the ones caused by the West Nile Virus (WNV)^2^ the St. Louis encephalitis virus and the Japanese encephalitis virus for example. In the case of WNV the virus originates in tropical regions and is carried over to other, less warmer, areas by infected birds through migration. By feeding on them, mosquitoes become carriers of the disease which then transmit the pathogen to humans. At all life stages *Culex* spp. are ectothermic and therefore climate sensitive. Studies have suggested that environmental changes, due to an increase in temperature and precipitation caused by global warming, have a significant impact on mosquito populations, facilitating their geographic spread into regions with more temperate climates further north and south^3–9^. A characteristic example is *Culex pipiens*. This is an ubiquitous mosquito species with a close association to humans and a worldwide distribution, inhabiting latitudes as high as Northern Europe and as low as the South Island of New Zealand^10,11^, which is a frequent vector of pathogens of both human and animal diseases^10,12^ . In an effort to assist public health authorities in devising appropriate health policies and vector control strategies an increasing number of mathematical models have been developed over the past years aimed at studying mosquito abundances and their dependence upon environmental factors^13–22^.

In this paper we perform a model free analysis of entomological data in the regional unit of Thessaloniki, an area which has seen an increase in the number of WNV reported cases in recent years^23^. The analysis is based on *Empirical Dynamic Modelling* (EDM). This is a data-driven method aimed at reconstructing the attractor manifold of a dynamical system from observations^24–27^ capable of assessing the dimensionality and degree of non-linearity of the underlying system. Using EDM it is possible to make short-term predictions of the system’s components^28–30^ and to infer its causal structure^31–33^. The technique has been applied successfully in various areas of research from paleontology^34–36^ and ecology^37–41^, to neuroscience^42–44^, climate studies^45–48^ and even solar weather prediction^49^. In a previous study, EDM was employed to uncover the environmental variables which drive mosquito abundance of the *Aedes* genus in French Polynesia based on the idea of *Convergent Cross Mapping*^31^. A drawback of this method is that it requires timeseries of a long length which, due to budgetary, time or other constraints, are usually not readily available. To overcome this challenge we apply the method of spatial-State Space Reconstruction (s-SSR)^32,72^, by combining spatial replicates of the variable in question into a composite timeseries, and infer causality from the cross-map skill between variables as a function of time delay between cause and effect. In contradistinction to previous applications we employ *vector correlation* metrics^73^ for measuring the quality of cross mapping which provide an enhancement over usual metrics as they are more robust under changes of the embedding dimension^74^.

## Results

### Forecasting yearly maximum abundances

**Figure 1:**
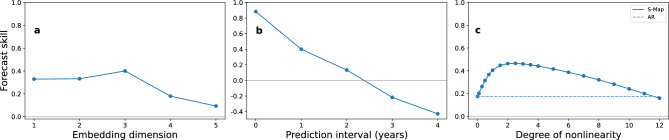
Leave-one-out forecast skill of predicted versus observed yearly differences in maximum mosquito abundance of a SP algorithm (**a**) as a function of the embedding dimension of the reconstructed state space for predictions made one year into the future and (**b**) as a function of the prediction interval for an embedding dimension $E=3$. (**c**) Forecast skill for predictions one year into the future between a local S-Map model and a global AR model as a function of the degree of nonlinearity of the system for an embedding dimension $E=3$.

As a first test case, we attempt to make out of sample forecasts of the maximum number of mosquitoes expected during the course of a year. The data consists of seven replicates of maximum abundances observed between the months of April and October from 2011 to 2021. Since the mean flight distance of *Culex* mosquitoes encountered in Europe is only of the order of a few hundred meters^50^, the large separation between locations (with a mean nearest neighbour distance of approximately 5.51 kilometers) ensures that the populations are isolated from one another. We therefore apply s-SSR (see Empirical Dynamic Modelling in Methods) to construct a composite state-space. To compensate for annual trends in the data, we first-difference each replicate separately to obtain yearly changes in maximum abundances. To determine the best embedding dimension for making predictions, we run a Simplex Projection (SP) algorithm (see Simplex Projection in Methods) with data from 2012 to 2019 as the input and perform a leave-one-out cross validation method to predict next year’s abundance between 2013 and 2020. In Fig. 1a we present the forecast skill of the SP algorithm, as a function of dimension *E*. We observe that the forecast skill displays a peak at an embedding dimension $E=3$. For this dimension the forecast skill was higher than $97\%$ of a 1000 randomly generated time-series using Ebisuzaki’s method^51^ (Table S1 in Supplementary). Projecting further into the future reveals the chaotic behaviour of the system with a decrease in forecast skill and a complete lack in predictability after two years (Fig. 1b). Comparing the forecast skill of a local S-Map model (see S-Map in methods) with that of a global autocorrelation (AR) model of the same dimension as a function of the degree of non-linearity (Fig. 1c) we observe the non-linear nature of the composite time-series with a peak at $\theta \simeq 2.5$, larger $97\%$ of the time when compared to a thousand random surrogates.

**Figure 2:**
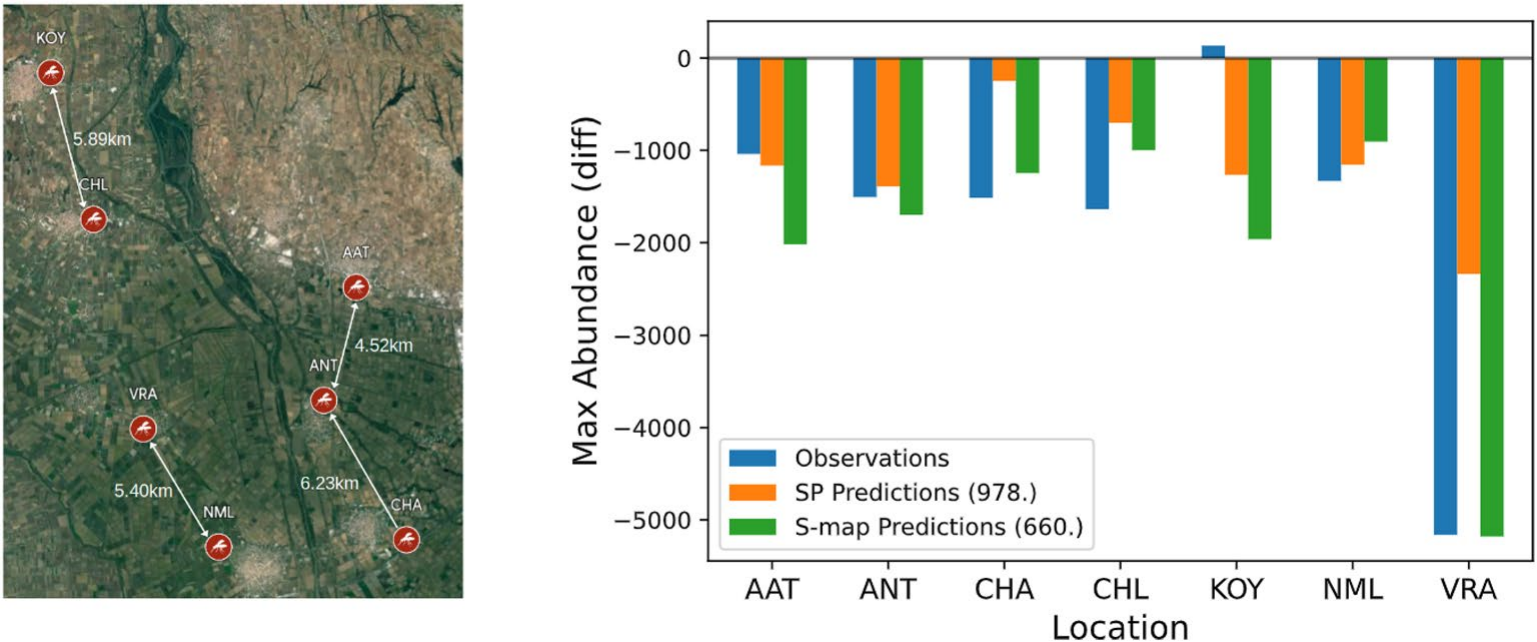
Left: Locations of spatial replicates and closest neighbour distance between sampling stations ANT, AAT, CHA, CHL, KOY, NML and VRA in kilometers (maps made with Google My Maps, Imagery $\copyright$2024 TerraMetrics). Right: SP and S-Map predictions of the difference in the maximum number of mosquitoes expected in 2021 compared to 2020 for $E=3$ and $\theta =2.5$. The mean absolute error between observations and predictions, is indicated in the labels.

Using the optimal values of *E* and $\theta$ determined above we now make out-of-sample forecasts of the maximum number of mosquitoes expected in 2021 compared to 2020 for each location. In Fig. 2 we present the predictions of the SP and S-Map algorithms. Both algorithms predict a decrease in the maximum number of mosquitoes expected at each location. Apart from one station (KOY) where a slight increase was actually observed, this was indeed the trend. We observe that the quality of predictions of the S-Map algorithm is better than those given by the SP algorithm with a mean absolute error of 420 and 904 respectively for the stations where prediction and observation match in trend. Excluding KOY from the library and retraining the algorithms with the same parameters shows a slight improvement in predictions with a mean absolute error of 809 for the SP and 403 for the S-Map algorithm, although in this case the optimum degree of non-linearity is found to be equal to $\theta \simeq 4.5$ (Fig. S1 in Supplementary). Running the algorithm again with the corrected degree increases the mean absolute error to 507 which is worse than that for $\theta \simeq 2.5$ but still better than the predictions obtained from the SP algorithm (Fig. S2 in Supplementary).

### Causal analysis of environmental effects on mosquito abundance

**Figure 3:**
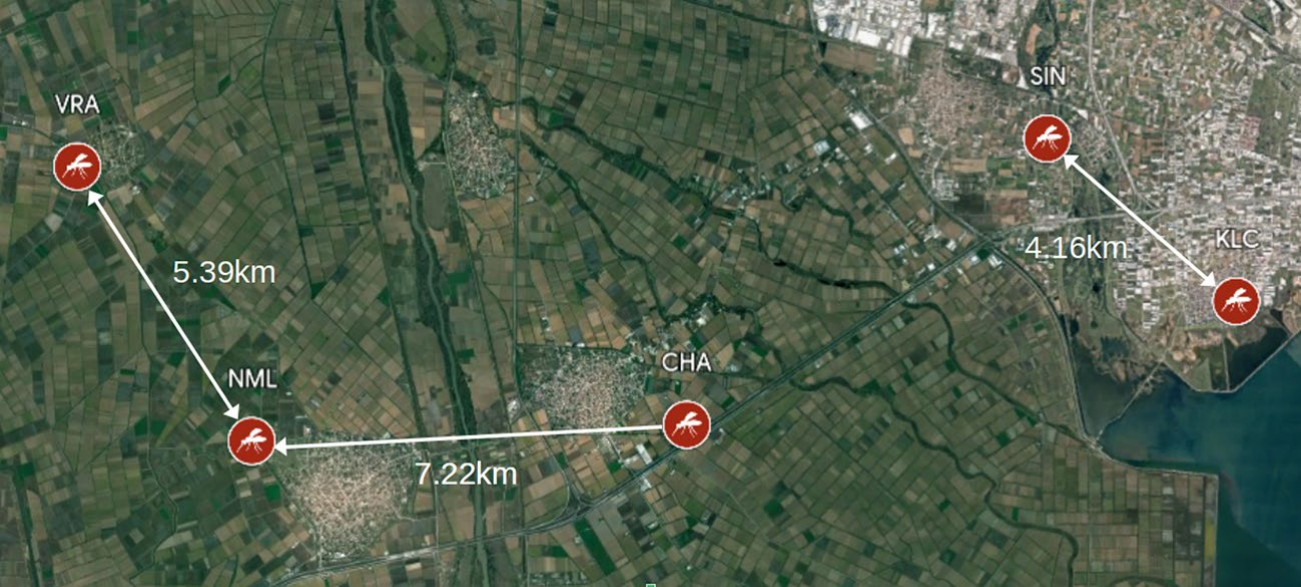
Locations of spatial replicates, CHA, KLC, NML, SIN, VRA and closest neighbour distance in kilometers for the causal analysis of environmental effects (maps made with Google My Maps, Imagery $\copyright$2024 TerraMetrics).

As a second test case we perform a causal analysis of environmental effects on mosquito populations. From the full set of possible sampling stations (see Data description in Methods), we select a subset of five replicates for which weekly abundances, sampled concurrently from June to September of 2012, were available with 17 consecutive weeks of entries per station (Fig. 3). Once again the large distance between locations (with a mean nearest neighbour distance of 5.59 kilometers) allows us to assume that the stations are independent from each other and we can therefore use s-SSR to construct from them a composite state-space. The environmental variables considered in the causal analysis, include the day mean of the *Land Surface Temperature* (LST), the accumulated rainfall one week before the date of placement (Rain), the mean hourly magnitude of wind (Wind), the *Normalized Difference Vegetation Index* (NDVI), the *Normalized Difference Moisture Index* (NDMI) and the *Normalized Difference Water Index* (NDWI) at each location. In order to increase the density of points in the reconstructed state space and improve the quality of the analysis, min-max normalization for each replicate is performed separately.

**Figure 4:**
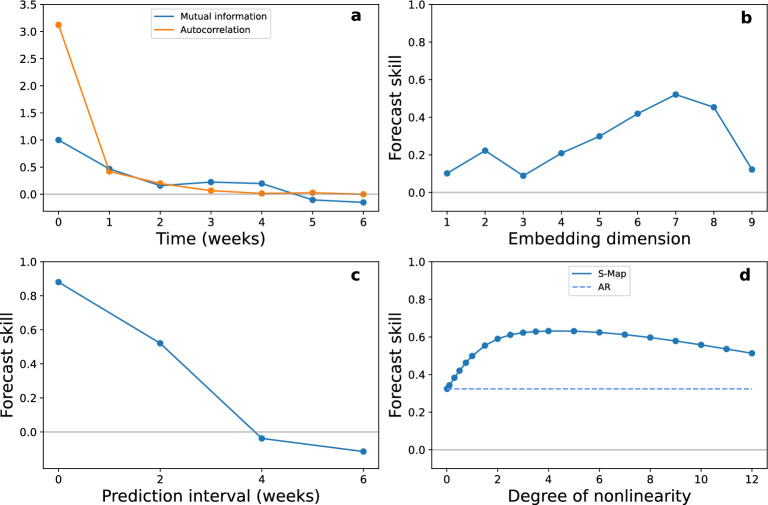
(**a**) Autocorrelation and mutual information of daily mosquito abundance as a function of time. (**b**) Leave-one-out forecast skill of SP algorithm as a function of the embedding dimension of the reconstructed state space for predictions two weeks into the future and (**c**) as a function of the prediction interval for an embedding dimension $E=7$. (**d**) Leave-one-out forecast skill as a function of the degree of non-linearity of a local S-Map model versus a global AR model for predictions two weeks into the future and an embedding dimension $E=7$.

Taking into consideration the developmental cycle of *Culex* mosquitoes, which lasts approximately two weeks, we predict with the help of the SP algorithm the state of the system two weeks into the future in order to determine the best embedding dimension. This choice of prediction interval coincides with the first local minimum of the mutual information and is also close to zero for the autocorrelation (Fig. 4a). In panel b of Fig. 4 we present the forecast ability between observed and predicted values of a leave-one-out bootstrap method. We observe that the forecast skill peaks at an embedding dimension $E=7$, which is higher than 99% of a 1000 randomly generated time-series using Ebisuzaki’s method (Table S2 in Supplementary). Forecasting further into the future shows a rapid decline in forecast skill (Fig. 4c) with a complete loss of predicting power after four weeks, a behaviour characteristic of a chaotic time-series. The system displays a non-linear behaviour, since the predictions of a local S-Map algorithm are more correlated to their corresponding observed values compared to the predictions of an AR method (Fig. 4d), with a peak at $\theta \simeq 4$, higher than $97\%$ of a 1000 surrogate time-series. Although the choice for the best embedding parameters is based on making predictions over a period of two weeks, the reconstructed manifolds contain time-lagged vectors with a lag of only $\tau =1$ week. The reason for this choice is the presence of different periods in the dynamics. Mosquito populations are expected to influence each other every fortnight while changes in abundances due to environmental effects can occur on a weekly basis^52^. Performing the above analysis with time-lagged vectors with $\tau =2$ weeks instead of one we find that the appropriate embedding dimension in this case has changed and is now equal to $E=4$. Nonetheless the characteristic behaviour of the time series when making predictions further into the future and its non-linearity remain qualitatively the same (Fig. S3 in Supplementary). This is consistent with studies which suggest that what is ultimately important for an EDM analysis is not the value of the embedding dimension *E* or of the time-delay $\tau$ separately bur rather the value of the embedding time-window^53–57^ $T_w = (E-1)\tau$ which in both cases here remains constant and equal to $T_w=6$.

Causality is now inferred by cross-mapping the reconstructed manifolds of each environmental variable with the one obtained from the data on mosquito abundance as a function of the prediction interval (see Cross mapping causal analysis in Methods). In Table 1 we display the average cross-map skill of a vector based correlation metric (See Correlation between random vectors in Methods) for predictions up to one month into the past and the future (for the forecast skill of each mapping as a function of prediction interval, used to calculate the averages, see Fig. S4 in Supplementary). From the positive values we detect LST, Rain and Wind as the main causal factors of daily mosquito abundance, with a mean peak in absolute prediction interval of half a week for temperature and one week for both precipitation and wind (Fig. S5 in Supplementary).

### Causal interaction of neighbouring populations

**Figure 5:**
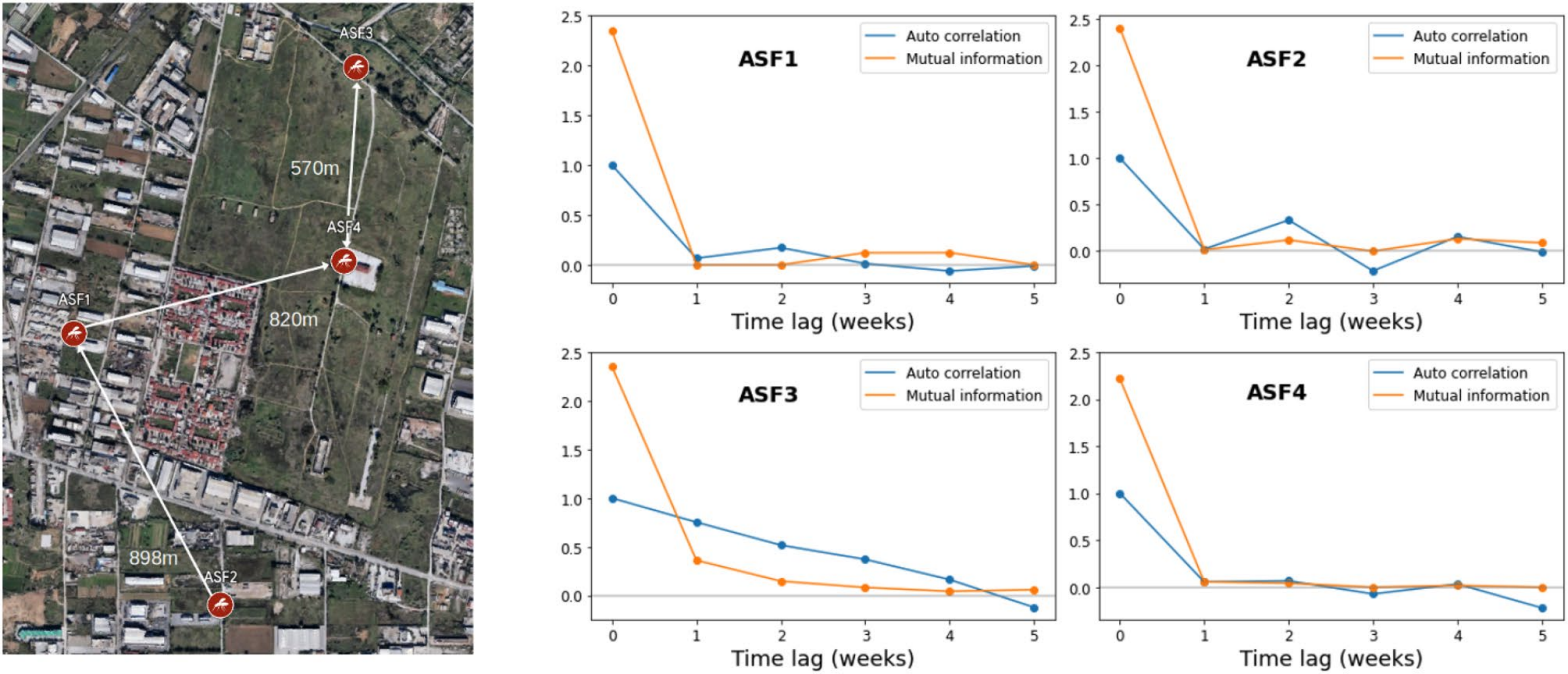
Left: Locations of replicates, ASF1,ASF2,ASF3,ASF4 and closest neighbour distance in meters (maps made with Google My Maps, Imagery $\copyright$2024 TerraMetrics). Right: Autocorrelation and mutual information of daily mosquito abundance as a function of time.

**Figure 6:**
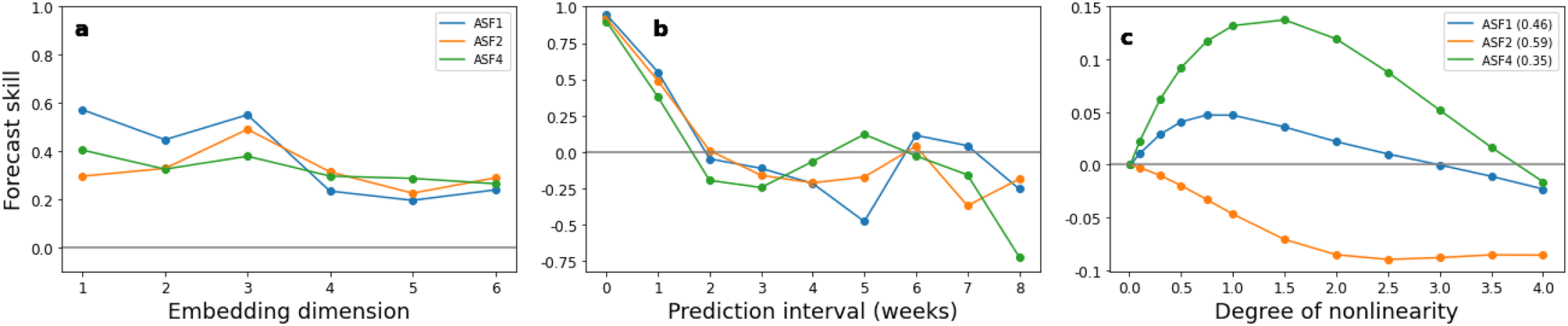
Leave-one-out forecast skill of SP algorithm (**a**) as a function of the embedding dimension of the reconstructed state space for predictions one week into the future and (**b**) as a function of the prediction interval for an embedding dimension $E=3$. (**c**) Difference in forecast skill for predictions one week into the future between a local S-Map model and a global AR model as a function of the degree of nonlinearity of the system for an embedding dimension $E=3$ (the forecast skill of the AR model is indicated in the labels).

We now carry out a causal analysis of neighbouring populations. The data in this case consists of four replicates of daily abundances sampled every week between the months of May and September of 2019 and 2020 with 19 and 21 consecutive weeks of entries respectively. In Fig. 5 we display, for each location, the autocorrelation and mutual information of the daily abundance as a function of time. For three out of the four locations (ASF1, ASF2, ASF4) the figure suggests that the choice for the best embedding parameters should be based on predictions made one week into the future. The remaining location (ASF3) shows a gradual decrease in autocorrelation indicating a difference in dynamics so is excluded from the subsequent analysis. To avoid annual trends, we make the data stationary by first-differencing each time-series to obtain weekly changes of abundances. Forecasting each replicate one week into the future we choose $E=3$ as the most suitable common embedding dimension (Fig. 6a). The observed forecast skill, for this dimension, compared to the one obtained from a thousand serially-correlated random timeseries generated with Ebisuzaki’s method was higher $99\%$, $88\%$ and $91\%$ of the time (Table S3 in Supplementary). Making predictions further into the future suggests that all three locations exhibit chaotic behaviour, with a decrease in forecast skill and an inability of making any predictions beyond two weeks (Fig. 6b). Running an S-Map analysis of the data as a function of the degree of nonlinearity of the time-series we could detect a non-linear behaviour in only two out of the three locations, ASF1 and ASF4, with $\theta \simeq 0.75$ ($89\%$ of the time higher) and $\theta \simeq 1.25$ ($96\%$ of the time higher) respectively.

In Table 2 we present the average cross-mapping skill of past and future predictions, between locations, for a vector correlation based metric (see Fig. S7 in Supplementary for the forecast skill of each mapping as a function of the prediction interval). We observe that ASF1 causally affects ASF2 with a mean peak in absolute prediction interval of approximately 4 weeks, while at the same time ASF2 affects location ASF4 with a mean peak in absolute prediction interval of 1 week. The latter location is also causally affected by ASF1. The mean absolute peak of 4.5 weeks in the prediction interval suggests in this case the existence of an indirect causal link between the two locations through ASF2.

## Discussion

The influence of human activity on the environment, either through increased invasion of animal habitats or changes in climate, is expected to result in an increase in the number of vector borne viral diseases such as yellow fever, Zika, chikungunya and West Nile Virus for example^1^. In light of this threat, techniques for accurately modeling and predicting the abundance of vectors carrying the virus, as in the case of mosquitoes, is important to public health authorities since it will allow them to anticipate possible future outbreaks and take appropriate preventive measures. Model free methods make no assumptions about the structure of the system under study and are therefore more general than their mechanistic counterparts. Applying EDM techniques on *Culex* data we were able to correctly predict the decreasing trend in the maximum number of mosquitoes observed during 2021 in a number of stations in the rural area of Thessaloniki. Using the same methodology it was also demonstrated that it is possible to deduce land surface temperature, the accumulated precipitation and the mean hourly wind as the key environmental causal factors driving mosquito abundance. This is consistent with other studies which indicate the same variables as the most important for the dynamics of mosquito populations with time lags similar to the ones reported here^7,14–17,58–65^. Temperature is a particularly important factor as it directly influences the mortality rate and life span of mosquitoes?^66–68^. Abundances peak at temperatures between 20 and 30^∘^C, corresponding to a short time in larval developments^2,12^, while high levels of mortality are found outside the 15 to 34^∘^C range. Performing a causal analysis on data in the industrial area of Thessaloniki we also detected interactions between neighboring populations. These were expected due to the short distance between the sampling stations (with a mean distance between closest neighbors of 714.5m) and can often be attributed to non-oriented dispersal of mosquitoes when searching for hosts, food, mates and places for oviposition or shelter^50,69^. Knowledge about interaction dynamics is useful information for the design of intervention strategies. Instead of chemically controlling a larger area for example one could focus attention locally only on those locations which causally affect mosquito abundance in others (such as ASF1 in our example) saving both valuable time and resources.

Though model free methods are advantageous, since they don’t suffer from any assumptions that need to be made in the construction of a mechanistic model, one of their main drawbacks is their need for long time-series with 30 or more consecutive observations (as a general rule of thumb). In order to apply these methods in ecological studies, where the available time-series are usually of a short length, it is necessary to combine several of them into a composite state-space using s-SSR. Any inferences based on these models will only therefore apply on a larger scale (depending on the mean closest neighbour distance between replicates) rather than on a local level. Our analysis on the causal effects of environmental factors, for example, suggested the possibility that vegetation, moisture and water content were also causal factors, but these were excluded as such on the basis that the average forecast skill of mapping the abundance manifold onto the manifold obtained from each of these environmental observables was less than zero in both past and future directions. The reason for this can be traced back to the composite nature of the reconstructed state space. Vegetation, moisture and water conditions differ considerably for each location while those for temperature, rainfall and wind are practically the same (Fig. S6 in Supplementary). The inability of making abundance predictions beyond a certain period, apart from the nonlinearity of population dynamics, is also related to the chaotic behaviour of weather. Due to Takens theorem the manifold reconstructed by time-lagged vectors of abundance observations is homeomorphic to the actual state-space of the system which includes environmental variables as degrees of freedom. Any limitations in the forecast ability of the later, with a horizon of approximately two weeks, will similarly affect the forecast ability of abundances. Despite these drawbacks EDM can serve as a useful guide in the construction of more accurate models. In all three cases considered here, each time-series exhibited a chaotic and (with the exception of series ASF2) non-linear behaviour. Comparing the autocorrelation and mutual information of Figs. 4 and 5 we also observe a change of period in the dynamics from two weeks to one week. Any realistic model should be capable to take these effects into account and qualitatively reproduce them. Future research on the relation between the dynamical characteristics (such as embedding dimension, the degree of non-linearity or the optimal time lag) of data used to train mechanistic models with the mathematical structure and choice of parameters of the latter, could possibly assist in that direction.

## Methods

### Data description

Data on mosquito abundances were obtained from EcoDevelopment SA. as part of the EarlY WArning System for Mosquito borne disease (EYWA) dataset, developed for the EuroGEO Action Group “Earth Observation for Epidemics of Vector-borne Diseases”, under the coordination of the National Observatory of Athens/BEYOND Centre of Earth Observation Research and Satellite Remote Sensing. The values of the *Normalized Difference Vegetation Index* (NDVI), the *Normalized Difference Moisture Index* (NDMI), the *Normalized Difference Water Index* (NDWI), the day mean of the *Land Surface Temperature* (LST), the accumulated rainfall one week before the date of placement (Rain) and the mean hourly magnitude of wind (Wind) at each location were obtained from the *Moderate Resolution Imaging Spectroradiometer* (MODIS) instrument aboard the Terra and Aqua satellites.

### Empirical dynamic modelling

EDM is based on Takens’s theorem which states that it is possible to construct an embedding of the manifold from lagged vectors of a single observed time-series *X*^24–27^

1 $${\mathbf{X}}(t) = \left\langle {X(t),X(t - \tau ), \ldots ,X(t - (E - 1)\tau )} \right\rangle$$

where $\tau$ is a *time-lag* which can be determined by the first zero of the autocorrelation function or the first local minimum of the mutual information^70,71^. Taking advantage of the topological structure of the reconstructed ‘shadow’ manifold it is possible to make predictions about the evolution of the system^28–30^ and infer its causal structure^31–33^. For series of a short length, as is usually the case in ecological studies, one can construct a composite state-space from spatial replicates using *spatial State Space Reconstruction*^32,72^ (s-SSR), discarding from the analysis any embedded vectors with overlap between their components.

### Simplex projection

Simplex projection^28^ is a *k-nearest neighbours* regression algorithm. For any observation $\textbf{X}(t)$ one can predict a feature $F(\textbf{X}(t))$ as the weighted average of its $E+1$ closest neighbours

2 $$\begin{aligned} \hat{F}(\textbf{X}(t))=\sum _{i=1}^{E+1}w_iF(\textbf{X}(t_i)), \end{aligned}$$

where

3 $$\begin{aligned} w_i=\frac{\exp \left[ -d_i/{{\bar{d}}}\right] }{ \sum _j\exp \left[ -d_j/{{\bar{d}}}\right] },\quad i=1\ldots ,E+1 \end{aligned}$$

is the weight of the i-th closest neighbour $\textbf{X}(t_i)$ to $\textbf{X}(t)$ with euclidean distance $d_i$, and ${\bar{d}}$ is the mean distance from all of its neighbours. Choosing $F(\textbf{X}(t_i))=X(t_i+T_p)$ in Eq. (2) one can employ the algorithm to make forecasts, by tracking where each neighbour will end up after $T_p$ time steps in the reconstructed state space. Making predictions one time step into the future it is possible to get an estimate for the best *embedding dimension* *E* by requiring the cross-validation between observed and predicted time-series to be maximized^28^. The same algorithm can also be used to distinguish between measurement error and chaos in the time-series. Making predictions further into the future for a chaotic system results in a sharp decrease in forecast skill compared to a noisy series for which the ability of making predictions as a function of the prediction interval $T_p$ remains relatively constant^28–30^.

### S-map

The S-Map algorithm can be used to test for non-linearity in a candidate time-series^30^. For every observation $\textbf{X}(t)$, an $E+1$ order autoregressive model is employed to forecast the value of the time-series $T_p$ time steps ahead as the scalar product of an $E+1$ dimensional vector of coefficients $\textbf{C}_t$ with $\textbf{X}(t)$

4 $$\begin{aligned} \hat{X}(t+T_p) = \textbf{C}_t\cdot \textbf{X}(t). \end{aligned}$$

The model is trained on a locally weighted set of lagged vectors

5 $$\begin{aligned} \left\{ \tilde{\textbf{X}}(t') = e^{-\theta d(t')/{\bar{d}}}{\textbf{X}}(t'),\quad t\ne t'\right\} ,\quad \theta \ge 0 \end{aligned}$$

where $d(t')$ is the euclidean distance of $\textbf{X}(t)$ from $\textbf{X}(t')$ and ${\bar{d}}$ is the mean distance. If $\theta >0$ then the training set is different for each prediction, with vectors closer to $\textbf{X}(t)$ contributing more to the model. This corresponds to a different vector of coefficients $\textbf{C}_t$ each time which can be calculated through a singular value decomposition or a least-squares approach. For $\theta =0$, $\textbf{C}_t$ is the same for every prediction and the model is global and equal to an autoregression algorithm of the same order. If the quality of predictions improves with the local model compared to the predictions obtained from the global model then the time-series can be considered to be non-linear with the degree of non-linearity represented by the parameter $\theta$.

### Cross mapping causal analysis

If *X* and *Y* are part of the same dynamical system then it is possible to infer whether a cause and effect relationship exists between the two variables by cross mapping their respective ‘shadow’ manifolds constructed from time lagged vectors of their observations^31–33^. By choosing $F(\textbf{X}(t_i))=Y(t_i+T_p)$ as a feature of the Simplex algorithm, it is possible to predict *Y* from *X* (in this case the prediction interval $T_p$ can take both positive as well as negative values). Causality can now be ascertained from the correlation between predicted and observed values as a function of $T_p$. If the mean correlation for positive values of $T_p$ is greater than that for negative values this means that past values of *X* are better at predicting future values of *Y* so it can be inferred that the former variable causally affects the latter, $X\prec Y$. If on the other hand the mean correlation for negative values of $T_p$ is greater that that for positive values then $Y\prec X$.

**Table 1:** Average cross-map skill of past ($T_p\le 0$) and future ($T_p\ge 0$) predictions between weekly mosquito abundances and Land Surface Temperature (LST), accumulated rainfall one week before the date of placement (Rain), mean hourly magnitude of wind (Wind), Normalized Difference Vegetation Index (NDVI), Normalized Difference Moisture Index (NDMI) and Normalized Difference Water Index (NDWI) for a vector correlation based metric. The quantities in parentheses indicate the value of the prediction interval (in weeks) where the cross mapping is maximized.

	$T_p\le 0$	$T_p\ge 0$		$T_p\le 0$	$T_p\ge 0$
**Culex** xmap **LST**	0.37 $(0\text{ w})$	0.31	**Culex** xmap **NDVI**	$-0.02$ $(-3\text{ w})$	$-0.21$
**LST** xmap **Culex**	0.20	0.37 $(1\text{ w})$	**NDVI** xmap **Culex**	$-0.02$	0.11 $(3\text{ w})$
**Culex** xmap **Rain**	0.32 $(-1\text{ w})$	0.26	**Culex** xmap **NDMI**	$-0.02$ $(-3\text{ w})$	$-0.23$
**Rain** xmap **Culex**	0.45	0.49 $(1\text{ w})$	**NDMI** xmap **Culex**	$-0.17$	$-0.07$ $(4\text{ w})$
**Culex** xmap **Wind**	0.27 $(-2\text{ w})$	0.09	**Culex** xmap **NDWI**	$-0.13$ $(-4\text{ w})$	$-0.20$
**Wind** xmap **Culex**	0.29	0.33 $(0\text{ w})$	**NDWI** xmap **Culex**	0.18	0.18 $(0\text{ w})$

**Table 2:** Average cross-map skill of past ($T_p\le 0$) and future ($T_p\ge 0$) predictions of weekly changes in mosquito abundance between neighboring locations for a vector correlation based metric. The quantities in parentheses indicate the value of the prediction interval (in weeks) where the cross mapping is maximized.

	$T_p\le 0$	$T_p\ge 0$		$T_p\le 0$	$T_p\ge 0$
**ASF1** xmap **ASF2**	$-0.12$	0.03 $(5\text{ w})$	**ASF1** xmap **ASF4**	$-0.17$	0.00 $(3\text{ w})$
**ASF2** xmap **ASF1**	0.12 $(-3\text{ w})$	0.22	**ASF4** xmap **ASF1**	0.08 $(-6\text{ w})$	$-0.11$
**ASF2** xmap **ASF4**	$-0.19$	0.06 $(1\text{ w})$			
**ASF4** xmap **ASF2**	0.26 $(-1\text{ w})$	0.14			

### Correlation between random vectors

Setting $F(\textbf{X}(t_i))=\textbf{Y}(t_i+T_p)$ as a feature of the Simplex algorithm, it is possible to predict the full lagged vector of *Y*, not only its first component. In order to assess the quality of predictions $\hat{\textbf{Y}}$ in this case we employ the following linear correlation coefficient which is suitable for use with random vectors

6 $$\begin{aligned} \rho (\textbf{Y},\hat{\textbf{Y}})=\frac{\text{ tr }(\Sigma _{{\textbf{Y}}\hat{\textbf{Y}}})}{\text{ tr }(\sqrt{\Sigma _{\textbf{Y}\textbf{Y}}\Sigma _{\hat{\textbf{Y}}\hat{\textbf{Y}}}})} \end{aligned}$$

where $\Sigma _{\textbf{X}\textbf{Y}}$ denotes the covariance matrix between random vectors $\textbf{X}$ and $\textbf{Y}$. It is proven that the above definition, which is a measure of the mean euclidean distance between the two sets of vectors, satisfies all of the properties of Pearson’s correlation coefficient^73^ which it reduces to in the univariate case. Recently, it was shown that the use of vector metrics provides a marked improvement of the causal structure of a non-linear system over other metrics^74^.

### Supplementary Information

Supplementary Information.

## Data Availability

The data that support the findings of this study are available from Ecodevelopment S.A. but restrictions apply to the availability of these data, which were used under license for the current study, and so are not publicly available. Data are however available from the corresponding author upon reasonable request and with permission of Ecodevelopment S.A.
