# Author Correction: Empirical dynamic modelling and enhanced causal analysis of short-length *Culex* abundance timeseries with vector correlation metrics

**DOI:** 10.1038/s41598-024-62267-w

**Published:** 2024-05-23

**Authors:** Nikos Kollas, Sandra Gewehr, Ioannis Kioutsioukis

**Affiliations:** 1 Department of Physics, University of Patras, 26504 Patras, Greece; 2 Ecodevelopment S.A., 57010 Filyro, Greece

Correction to: *Scientific Reports* 10.1038/s41598-024-54054-4, published online 13 February 2024

The original version of this Article contained an error in the name of Nikos Kollas, Sandra Gewehr and Ioannis Kioutsioukis, which were given as Kollas Nikos, Gewehr Sandra and Kioutsioukis Ioannis.

The original Article has been corrected.

