# Supplementary material for: Empirical dynamic modelling and enhanced causal analysis of short-length Culex abundance timeseries with vector correlation metrics
Source: Sci Rep. 2024 Feb 13;14:3597. doi: 10.1038/s41598-024-54054-4 (PMC10864305; doi:10.1038/s41598-024-54054-4)
Supplement: Supplementary file 1 — Supplementary Information. [file 41598_2024_54054_MOESM1_ESM.pdf]

Empirical dynamic modelling and enhanced causal analysis of short-length *Culex* abundance timeseries with vector correlation metrics

Kollas Nikolaos[1], **Gewehr Sandra**[2], **and Kioutsioukis Ioannis**[1*]

[1]Department of Physics, University of Patras, 26504, Patras, Greece
[2]Ecodevelopment S.A., 57010, Filyro, Greece
[*]Corresponding Author e-mail: kioutio@upatras.gr

Forecasting yearly maximum abundances

**E**	**1**	**2**	**3**	**4**	**5**
**p**	0.08	0.12	0.03	0.37	0.44

Table S1. Percentage of randomly generated serially correlated composite surrogates for which the forecast ability was greater than or equal to the one observed as a function of the embedding dimension.

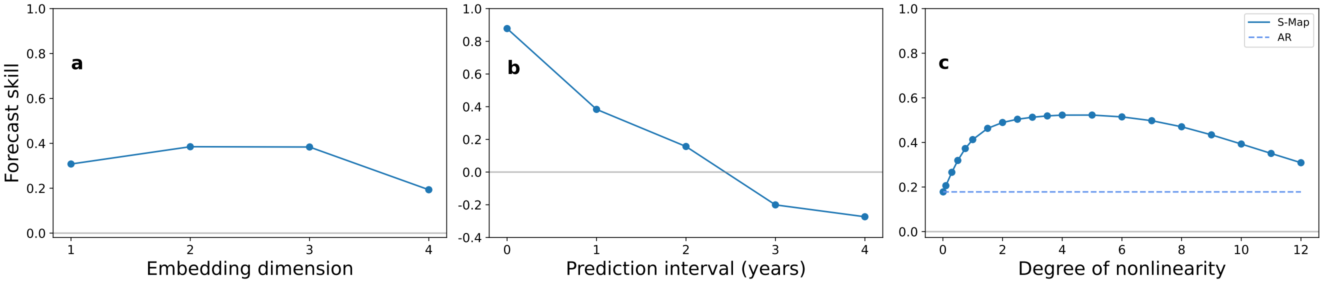

Figure S1. Leave-one-out forecast skill of predicted versus observed yearly changes in maximum mosquito abundance of a SP algorithm **a)** as a function of the embedding dimension of the reconstructed state space one year into the future and **b)** as a function of the prediction interval for an embedding dimension $E = 3$. **c)** Forecast skill for predictions one year into the future between a local S-Map model and a global AR model as a function of the degree of nonlinearity of the system for an embedding dimension $E = 3$.

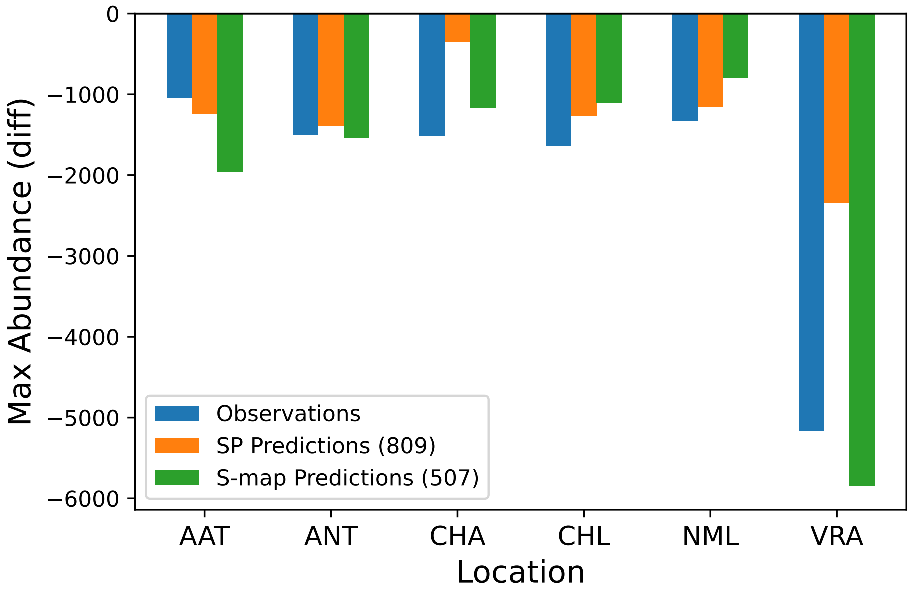

Figure S2. SP and S-Map predictions of the difference in the maximum number of mosquitoes expected in 2021 compared to 2020 for $E = 3$ and $\theta = 4.5$. The mean absolute error between observations and predictions, is indicated in the labels.

Causal analysis of environmental effects on mosquito abundance

**E**	**1**	**2**	**3**	**4**	**5**	**6**	**7**	**8**	**9**
**p**	0.3	0.1	0.35	0.14	0.09	0.03	0.01	0.08	0.61

Table S2. Percentage of randomly generated serially correlated surrogates for which the forecast ability two weeks into the future was greater than or equal to the one observed as a function of the embedding dimension.

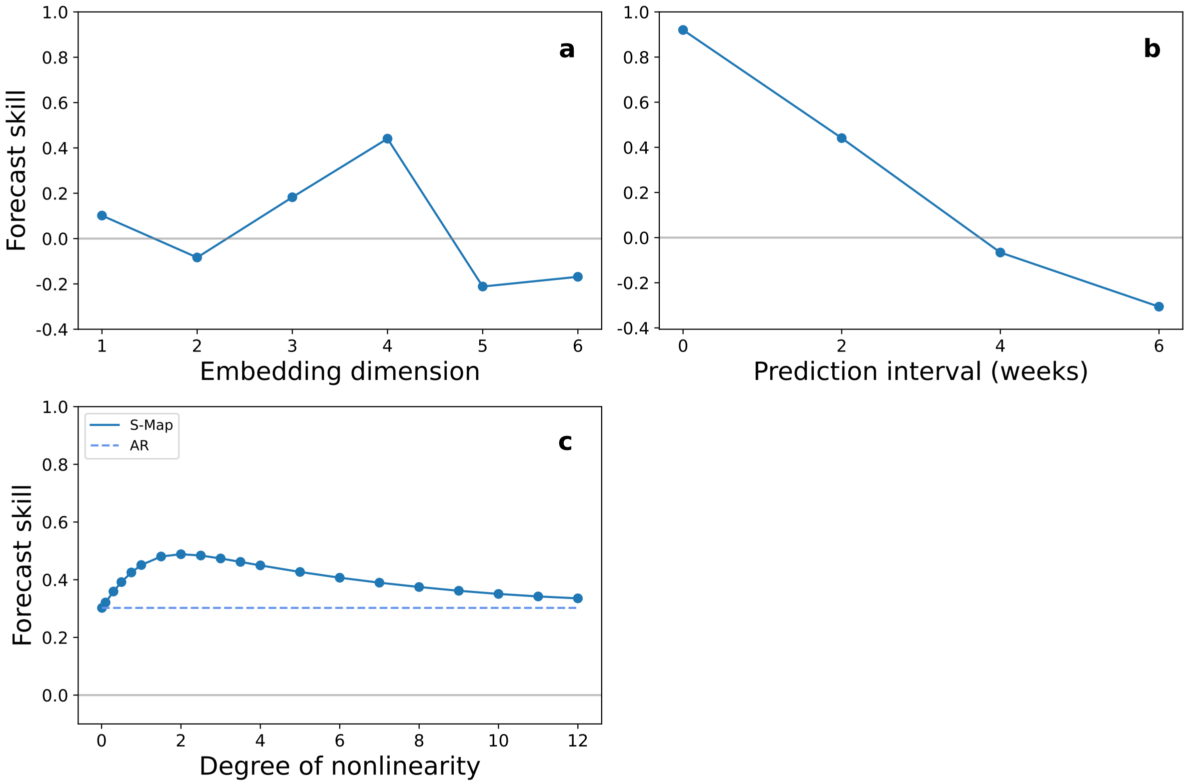

Figure S3. **a)** Leave-one-out forecast skill of Simplex projection algorithm for predicting daily mosquito abundances two weeks into the future as a function of the embedding dimension of the reconstructed state space of time-lagged vectors with a time lag $\tau = 2$ weeks and **b)** as a function of the prediction interval for an embedding dimension $E = 4$. **c)** Forecast skill of a local S-Map model versus a global AR model for an embedding dimension $E = 4$ as a function of the degree of non-linearity.

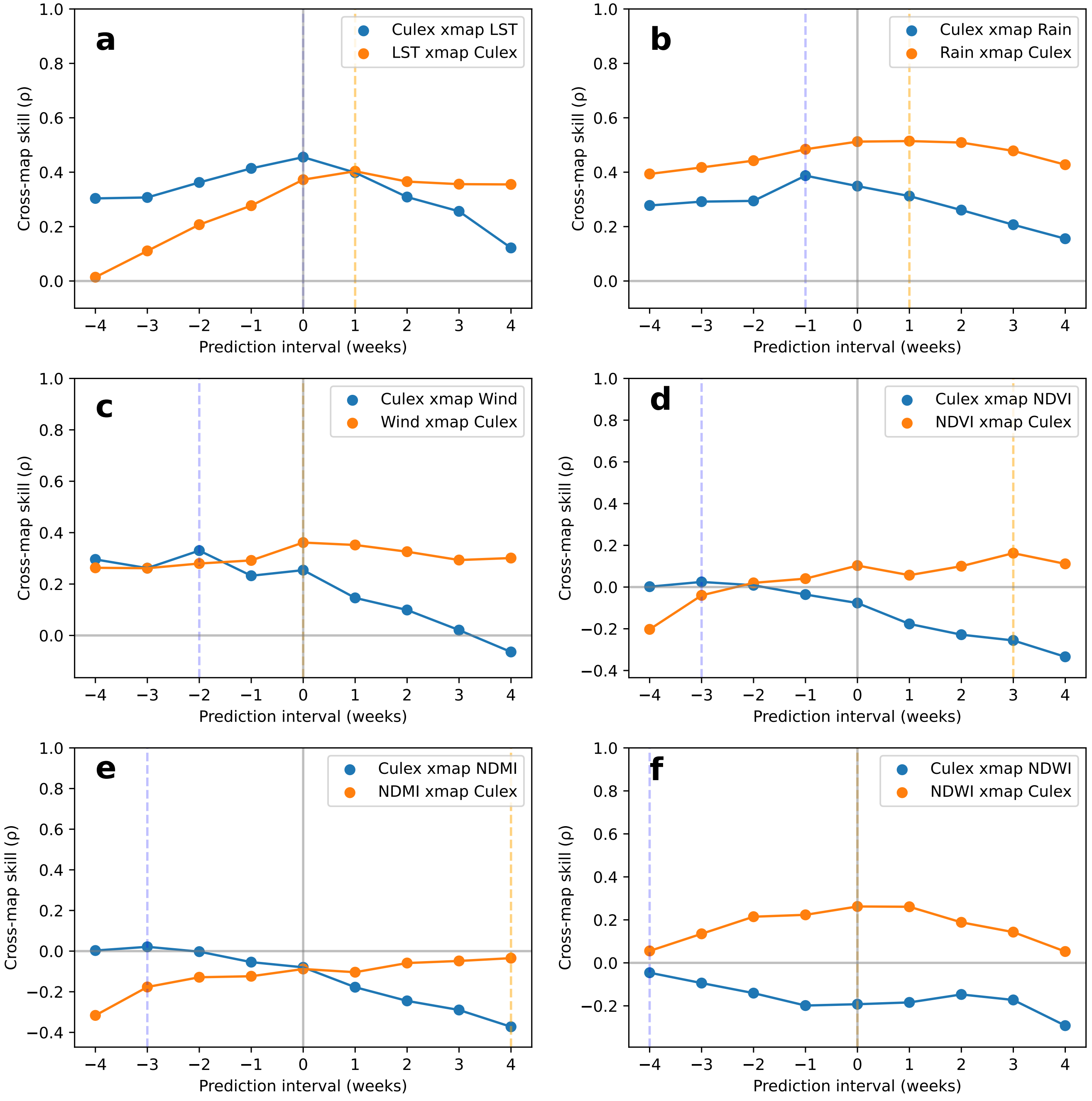

Figure S4. Vector correlation based cross-map skill between the mosquito abundance (Culex) manifold and **a)** the day mean of the land surface temperature (LST), **b)** the accumulated rainfall one week before date of placement (Rain), **c)** the mean hourly magnitude of wind (Wind), **d)** the normalized difference vegetation index (NDVI), **e)** the normalized difference moisture index (NDMI) and **f)** the normalized difference water index (NDWI) manifolds as a function of the prediction interval.

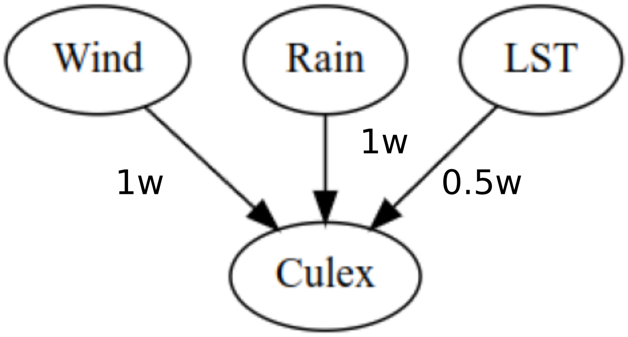

Figure S5. Causal network of environmental effects on daily mosquito abundance based on the vector correlation between observed and predicted vectors of the reconstructed lagged state space. The numbers indicate the mean values of the absolute prediction interval (in weeks) for which the cross mapping was maximized.

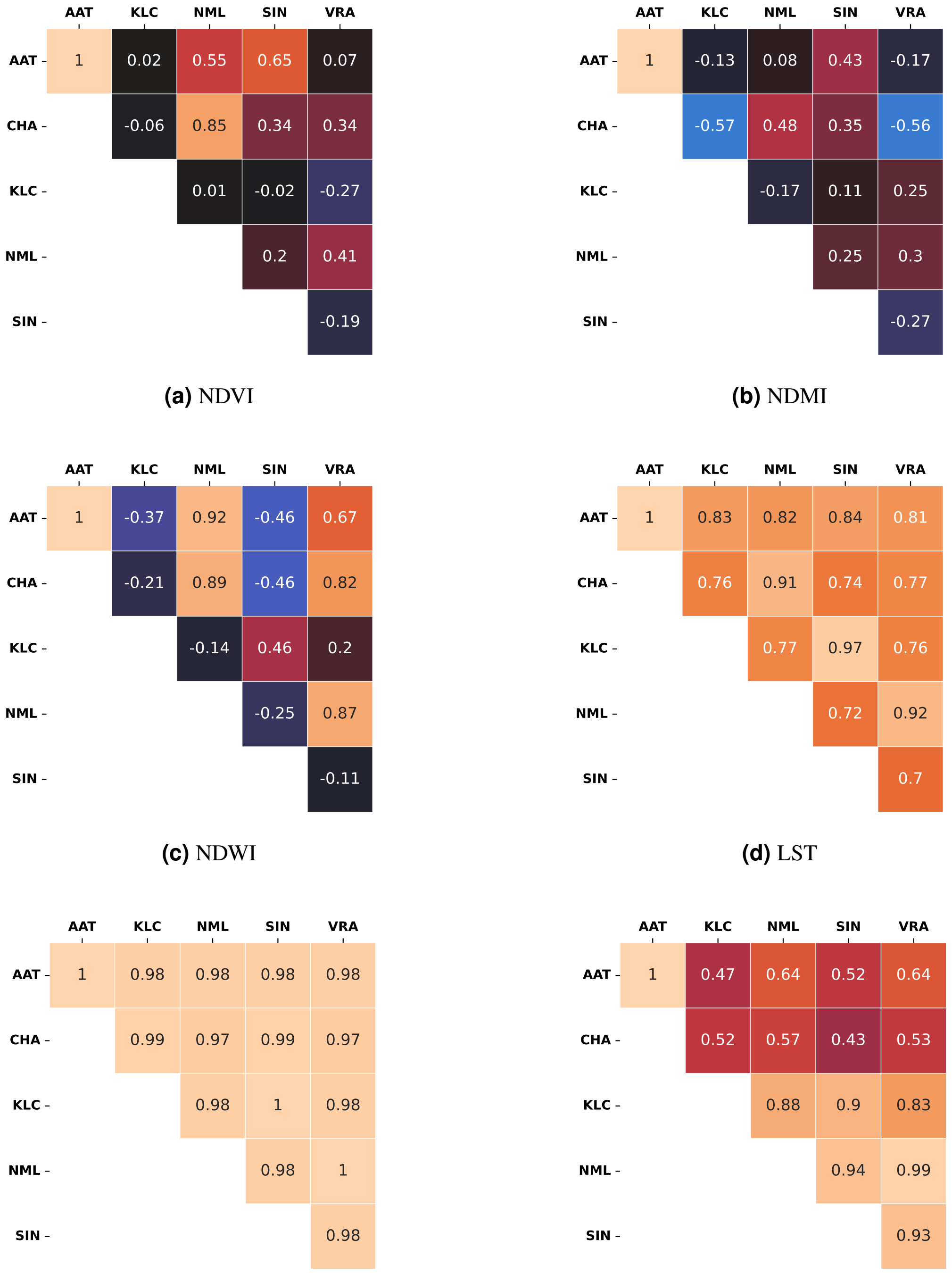

(a) NDVI

(b) NDMI

(c) NDWI

(d) LST

(e) Rain

(f) Wind

Figure S6. Correlation matrix of **a)** the normalized difference vegetation index (NDVI), **b)** the normalized difference moisture index (NDMI) **c)** the normalized difference water index (NDWI) **d)** the day mean of the land surface temperature (LST) **e)** the accumulated rainfall one week before date of placement (Rain) and **f)** the mean hourly magnitude of wind (Wind) between spatial replicates

Causal interaction of neighbouring populations

E	1	2	3	4	5	6
**ASF1**	0.01	0.10	0.01	0.31	0.55	0.55
**ASF2**	0.71	0.63	0.12	0.54	0.55	0.29
**ASF4**	0.10	0.23	0.09	0.14	0.18	0.20

Table S3. Percentage of randomly generated serially correlated surrogates for which the forecast ability was greater than or equal to the one observed as a function of the embedding dimension.

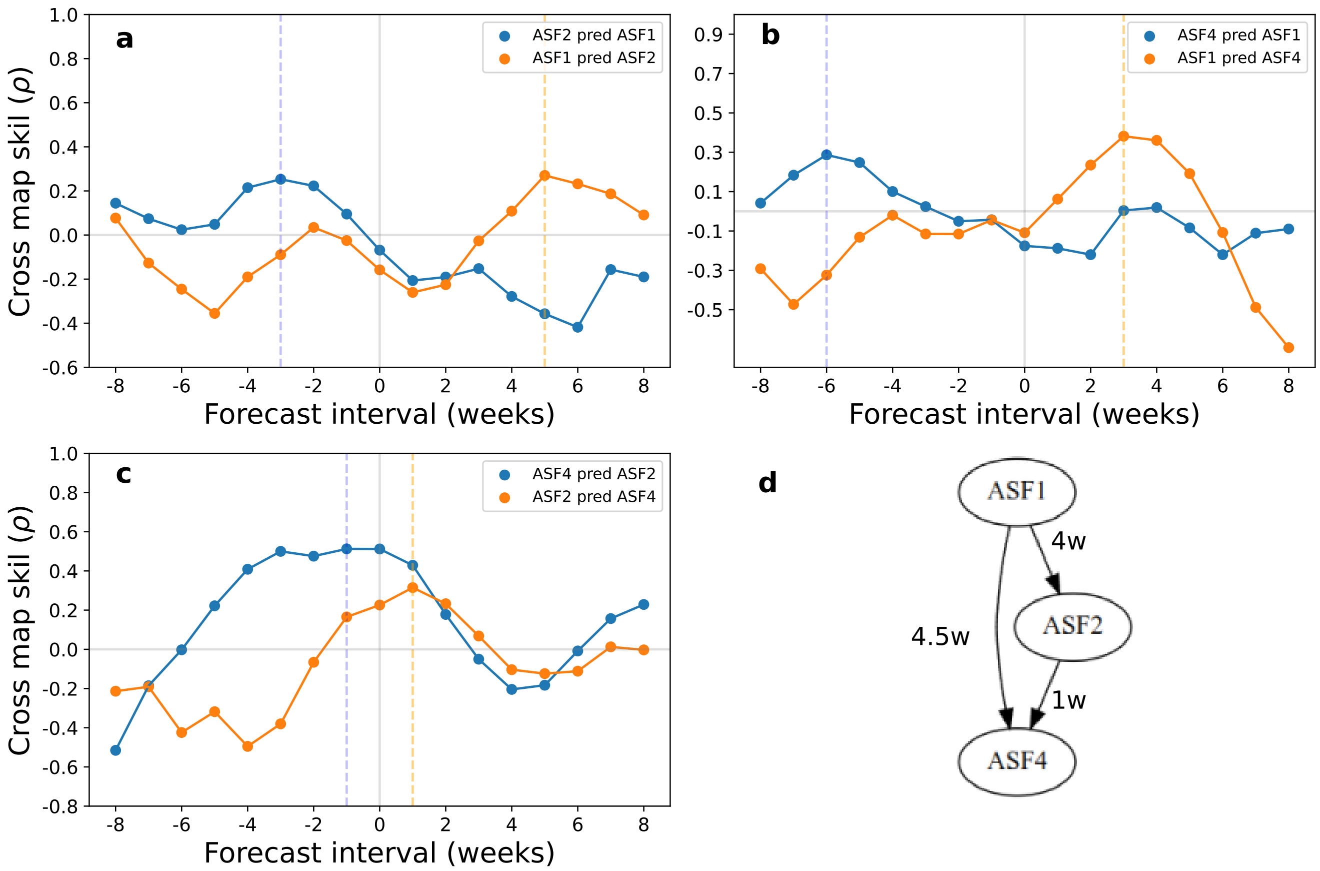

Figure S7. Vector correlation based cross-map skill of weekly changes in mosquito abundance between neighbouring locations **a)** ASF1 vs ASF2 **b)** ASF1 vs ASF4 and **c)** ASF2 vs ASF4 as as a function of the prediction interval. **d)** Resulting causal network.
